# 9-(4-Fluoro­phen­oxy­carbon­yl)-10-methyl­acridinium trifluoro­methane­sulfonate

**DOI:** 10.1107/S1600536810039231

**Published:** 2010-10-09

**Authors:** Damian Trzybiński, Karol Krzymiński, Jerzy Błażejowski

**Affiliations:** a Faculty of Chemistry, University of Gdańsk, J. Sobieskiego 18, 80-952 Gdańsk, Poland

## Abstract

In the crystal structure of the title compound, C_21_H_15_FNO_2_^+^·CF_3_SO_3_^−^, the cations form inversion dimers through C—H⋯O, C—F⋯π and π–π inter­actions. These dimers are further linked by π–π inter­actions. The cations and anions are connected through C—H⋯O, C—F⋯π and S—O⋯π inter­actions. The acridine and benzene ring systems are oriented at a dihedral angle of 74.1 (1)°. The carboxyl­ate group is twisted at an angle of 4.4 (1)° relative to the acridine skeleton. The mean planes of the adjacent acridine moieties are parallel or inclined at an angle of 55.4 (1)° in the crystal structure.

## Related literature

For general background to the chemiluminogenic properties of 9-phen­oxy­carbonyl-10-methyl­acridinium trifluoro­methane­sulfonates, see: Brown *et al.* (2009 ▶); King *et al.* (2007 ▶); Rak *et al.* (1999 ▶); Roda *et al.* (2003 ▶); Zomer & Jacquemijns (2001 ▶). For related structures, see: Sikorski *et al.* (2005 ▶); Trzybiński *et al.* (2010 ▶). For inter­molecular inter­actions, see: Bianchi *et al.* (2004 ▶); Dorn *et al.* (2005 ▶); Hunter *et al.* (2001 ▶); Novoa *et al.* (2006 ▶). For the synthesis, see: Sato (1996 ▶); Sikorski *et al.* (2005 ▶).
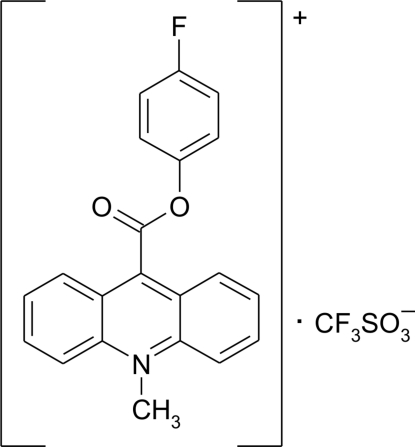


## Experimental

### 

#### Crystal data

C_21_H_15_FNO_2_^+^·CF_3_SO_3_^−^
*M* *_r_* = 481.41
Monoclinic, 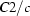

*a* = 20.854 (3) Å
*b* = 7.8092 (12) Å
*c* = 25.690 (4) Å
β = 100.893 (15)°
*V* = 4108.2 (11) Å^3^
*Z* = 8
Mo *K*α radiation
μ = 0.23 mm^−1^
*T* = 295 K
0.38 × 0.29 × 0.05 mm

#### Data collection

Oxford Diffraction Gemini R Ultra Ruby CCD diffractometer
Absorption correction: multi-scan (*CrysAlis RED*; Oxford Diffraction, 2008 ▶) *T*_min_ = 0.676, *T*_max_ = 0.985
15588 measured reflections
3634 independent reflections
1978 reflections with *I* > 2σ(*I*)
*R*_int_ = 0.045

#### Refinement

*R*[*F* ^2^ > 2σ(*F* ^2^)] = 0.042
*wR*(*F* ^2^) = 0.117
*S* = 0.91
3634 reflections
299 parameters
H-atom parameters constrained
Δρ_max_ = 0.17 e Å^−3^
Δρ_min_ = −0.25 e Å^−3^

### 

Data collection: *CrysAlis CCD* (Oxford Diffraction, 2008 ▶); cell refinement: *CrysAlis RED* (Oxford Diffraction, 2008 ▶); data reduction: *CrysAlis RED*; program(s) used to solve structure: *SHELXS97* (Sheldrick, 2008 ▶); program(s) used to refine structure: *SHELXL97* (Sheldrick, 2008 ▶); molecular graphics: *ORTEP-3* (Farrugia, 1997 ▶); software used to prepare material for publication: *SHELXL97* and *PLATON* (Spek, 2009 ▶).

## Supplementary Material

Crystal structure: contains datablocks global, I. DOI: 10.1107/S1600536810039231/ng5037sup1.cif

Structure factors: contains datablocks I. DOI: 10.1107/S1600536810039231/ng5037Isup2.hkl

Additional supplementary materials:  crystallographic information; 3D view; checkCIF report

## Figures and Tables

**Table 1:** Hydrogen-bond geometry (Å, °)

*D*—H⋯*A*	*D*—H	H⋯*A*	*D*⋯*A*	*D*—H⋯*A*
C1—H1⋯O17^i^	0.93	2.49	3.299 (3)	146
C4—H4⋯O27	0.93	2.46	3.185 (3)	134
C5—H5⋯O27^ii^	0.93	2.53	3.200 (4)	130
C22—H22⋯O29^iii^	0.93	2.54	3.399 (3)	153

Symmetry codes: (i) 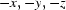; (ii) 
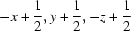
; (iii) 
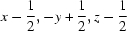
.

**Table 2:** C—F⋯π and S—O⋯π inter­actions (Å,°) *Cg*1 and *Cg*2 are the centroids of the C9/N10/C11–C14 and C1–C4/C11/C12 rings, respectively.

*X*	*I*	*J*	*I*⋯*J*	*X*⋯*J*	*X*—*I*⋯*J*
C21	F24	*Cg*2^i^	3.870 (2)	3.616 (3)	69.12 (12)
C30	F33	*Cg*2^iv^	3.835 (2)	4.951 (4)	143.41 (19)
S26	O29	*Cg*1^ii^	3.646 (2)	5.055 (15)	170.66 (13)

Symmetry codes: (i) −*x*, −*y*, −*z*; (ii) −*x* + 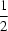, *y* + 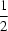, −*z* + 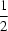; (iv) −*x*, *y*, −*z* + 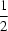.

**Table 3:** π–π inter­actions (Å,°) *Cg*1, *Cg*2, *Cg*3 and *Cg*4 are the centroids of the C9/N10/C11–C14, C1–C4/C11/C12, C5–C8/C13/C14 and C18–C23 rings, respectively. *CgI*⋯*CgJ* is the distance between ring centroids. The dihedral angle is that between the planes of the rings *I* and *J*. *CgI*_Perp is the perpendicular distance of *CgI* from ring *J*. *CgI*_Offset is the distance between *CgI* and perpendicular projection of *CgJ* on ring *I*.

*I*	*J*	*CgI*⋯*CgJ*	Dihedral angle	*CgI*_Perp	*CgI*_Offset
1	4^v^	3.572 (2)	5.04 (11)	3.408 (1)	1.089 (2)
2	4^i^	3.856 (2)	4.29 (13)	3.596 (2)	1.392 (2)
3	4^v^	3.898 (2)	4.66 (12)	3.380 (2)	1.942 (2)
4	1^v^	3.572 (2)	5.04 (11)	3.472 (1)	0.839 (2)
4	2^i^	3.856 (2)	4.29 (13)	3.502 (1)	1.614 (2)
4	3^v^	3.898 (2)	4.66 (12)	3.483 (1)	1.750 (2)

Symmetry codes: (i) −*x*, −*y*, −*z*; (v) −*x*, −*y* + 1, −*z*.

**Fig. 1.**
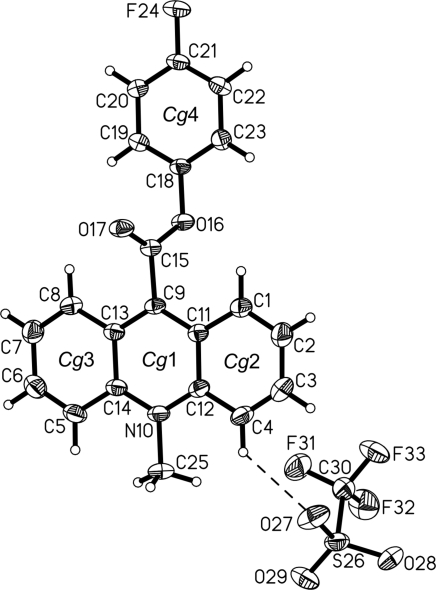
The molecular structure of the title compound showing the atom-labeling scheme. Displacement ellipsoids are drawn at the 25% probability level and H atoms are shown as small spheres of arbitrary radius. Cg1, Cg2, Cg3 and Cg4 denote the ring centroids. The C-H···O interaction is represented by dashed lines.

**Fig. 2.**
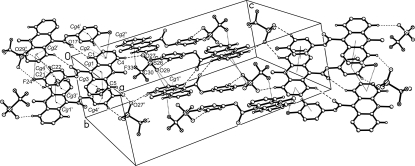
The arrangement of the ions in the crystal structure. The C-H···O interactions are represented by dashed lines, the C-F···π, S-O···π and π-π contacts by dotted lines. H atoms not involved in interactions have been omitted. [Symmetry codes: (i) -x, -y, -z; (ii) -x + 1/2, y + 1/2, -z + 1/2; (iii) x - 1/2, -y + 1/2, z - 1/2; (iv) -x, y, -z + 1/2; (v) -x, -y + 1, -z.]

## supplementary crystallographic information

### Comment

9-(Phenoxycarbonyl)-10-methylacridinium salts have long been known as chemiluminescent indicators or the chemiluminogenic fragments of chemiluminescent labels widely used in assays of biologically and environmentally important entities such as antigens, antibodies, enzymes or DNA fragments (Zomer & Jacquemijns, 2001; Roda *et al.*, 2003; King *et al.*, 2007; Brown *et al.*, 2009). The cations of these salts are oxidized with hydrogen peroxide in alkaline media, which produces light. It has been found that this process is accompanied by the removal of the phenoxycarbonyl fragment and the conversion of the remaining part of the molecules to electronically excited, light-emitting 10-methyl-9-acridinone (Rak *et al.*, 1999). The efficiency of chemiluminescence - crucial for analytical applications - is affected by the constitution of the phenyl fragment (Zomer & Jacquemijns, 2001). In the search for efficient chemiluminogens we undertook investigations on 9-(phenoxycarbonyl)-10-methylacridinium derivatives substituted in the phenyl fragment. Here we present the structure of 9-(4-fluorophenoxycarbonyl)-10-methylacridinium trifluoromethanesulfonate.

In the cation of the title compound (Fig. 1), the bond lengths and angles characterizing the geometry of the acridinium moiety are typical of acridine-based derivatives (Sikorski *et al.*, 2005; Trzybiński *et al.*, 2010). With respective average deviations from planarity of 0.0288 (3) Å and 0.0081 (3) Å, the acridine and benzene ring systems are oriented at a dihedral angle of 74.1 (1)°. The carboxyl group is twisted at an angle of 4.4 (1)° relative to the acridine skeleton. The mean planes of the adjacent acridine moieties are parallel (remain at an angle 0.0 (1)°) or inclined at an angle of 55.4 (1)° in the lattice.

In the crystal structure, the inversely oriented cations form dimers through multidirectional C-H···O (Table 1, Fig. 2), C-F···π (Table 2, Fig. 2) and π-π (Table 3, Fig. 2) interactions. These dimers are further linked by π-π (Table 3, Fig. 2) interactions. The adjacent cations (dimers) and anions are connected through C-H···O (Table 1, Fig. 2), C-F···π (Table 2, Fig. 2) and S-O···π  (Table 2, Fig. 2) interactions. The C-H···O interactions are of the hydrogen bond type (Bianchi *et al.* 2004; Novoa *et al.* 2006). C-F···π (Dorn *et al.*, 2005), S-O···π (Dorn *et al.*, 2005) and the π-π (Hunter *et al.*, 2001) interactions should be of an attractive nature. The crystal structure is stabilized by a network of these short-range specific interactions and by long-range electrostatic interactions between ions.

### Experimental

The compound was synthesized in two steps (Sikorski *et al.*, 2005). First, 9-(chlorocarbonyl)acridine, obtained by treating acridine-9-carboxylic acid with a tenfold molar excess of thionyl chloride, was esterified with 4-fluorophenol in anhydrous dichloromethane in the presence of *N*,*N*-diethylethanamine and a catalytic amount of *N*,*N*-dimethyl-4-pyridinamine (room temperature, 15h) (Sato, 1996). Second, the product - 4-fluorophenylacridine-9-carboxylate, purified chromatographically (SiO_2_, cyclohexane/ethyl acetate, 1/1 v/v) - was quaternarized with a fivefold molar excess of methyl trifluoromethanesulfonate dissolved in anhydrous dichloromethane. The crude 9-(4-fluorophenoxycarbonyl)-10-methylacridinium trifluoromethanesulfonate was dissolved in a small amount of ethanol, filtered and precipitated with 20 v/v excess of diethyl ether. Yellow crystals suitable for X-ray investigations were grown from absolute ethanol solution (m.p. 474-475 K).

### Refinement

H atoms were positioned geometrically, with C–H = 0.93 Å and 0.96 Å for the aromatic and methyl H atoms, respectively, and constrained to ride on their parent atoms with *U*_iso_(H) = *xU*_eq_(C), where *x* = 1.2 for the aromatic and *x* = 1.5 for the methyl H atoms.

### Crystal data

**Table:**

C_21_H_15_FNO_2_^+^·CF_3_SO_3_^−^	*F*(000) = 1968
*M**_r_* = 481.41	*D*_x_ = 1.557 Mg m^−^^3^
Monoclinic, *C*2/*c*	Mo *K*α radiation, λ = 0.71073 Å
Hall symbol: -C 2yc	Cell parameters from 3994 reflections
*a* = 20.854 (3) Å	θ = 3.0–24.9°
*b* = 7.8092 (12) Å	µ = 0.23 mm^−^^1^
*c* = 25.690 (4) Å	*T* = 295 K
β = 100.893 (15)°	Plate, yellow
*V* = 4108.2 (11) Å^3^	0.38 × 0.29 × 0.05 mm
*Z* = 8	

### Data collection

**Table:**

Oxford Diffraction Gemini R Ultra Ruby CCD diffractometer	3634 independent reflections
Radiation source: Enhanced (Mo) X-ray Source	1978 reflections with *I* > 2σ(*I*)
graphite	*R*_int_ = 0.045
Detector resolution: 10.4002 pixels mm^-1^	θ_max_ = 25.1°, θ_min_ = 3.1°
ω scans	*h* = −23→24
Absorption correction: multi-scan (*CrysAlis RED*; Oxford Diffraction, 2008)	*k* = −9→9
*T*_min_ = 0.676, *T*_max_ = 0.985	*l* = −30→30
15588 measured reflections	

### Refinement

**Table:**

Refinement on *F*^2^	Primary atom site location: structure-invariant direct methods
Least-squares matrix: full	Secondary atom site location: difference Fourier map
*R*[*F*^2^ > 2σ(*F*^2^)] = 0.042	Hydrogen site location: inferred from neighbouring sites
*wR*(*F*^2^) = 0.117	H-atom parameters constrained
*S* = 0.91	*w* = 1/[σ^2^(*F*_o_^2^) + (0.068*P*)^2^] where *P* = (*F*_o_^2^ + 2*F*_c_^2^)/3
3634 reflections	(Δ/σ)_max_ < 0.001
299 parameters	Δρ_max_ = 0.17 e Å^−^^3^
0 restraints	Δρ_min_ = −0.25 e Å^−^^3^

### Special details

**Table:**

Geometry. All esds (except the esd in the dihedral angle between two l.s. planes) are estimated using the full covariance matrix. The cell esds are taken into account individually in the estimation of esds in distances, angles and torsion angles; correlations between esds in cell parameters are only used when they are defined by crystal symmetry. An approximate (isotropic) treatment of cell esds is used for estimating esds involving l.s. planes.

### Fractional atomic coordinates and isotropic or equivalent isotropic displacement parameters (Å^2^)

**Table:**

	*x*	*y*	*z*	*U*_iso_*/*U*_eq_	
C1	0.03283 (13)	0.1193 (3)	0.11048 (10)	0.0600 (7)	
H1	0.0011	0.1023	0.0802	0.072*	
C2	0.02112 (15)	0.0689 (4)	0.15766 (12)	0.0797 (9)	
H2	−0.0186	0.0186	0.1602	0.096*	
C3	0.06910 (18)	0.0924 (5)	0.20305 (12)	0.0860 (10)	
H3	0.0608	0.0560	0.2356	0.103*	
C4	0.12683 (16)	0.1660 (4)	0.20108 (10)	0.0707 (8)	
H4	0.1578	0.1792	0.2320	0.085*	
C5	0.27041 (13)	0.4437 (4)	0.09888 (12)	0.0660 (7)	
H5	0.3020	0.4586	0.1293	0.079*	
C6	0.28130 (13)	0.5020 (4)	0.05259 (13)	0.0733 (8)	
H6	0.3205	0.5572	0.0514	0.088*	
C7	0.23522 (14)	0.4824 (4)	0.00556 (11)	0.0698 (8)	
H7	0.2435	0.5262	−0.0262	0.084*	
C8	0.17902 (13)	0.3997 (3)	0.00688 (9)	0.0568 (7)	
H8	0.1488	0.3852	−0.0244	0.068*	
C9	0.10650 (12)	0.2530 (3)	0.05781 (9)	0.0441 (6)	
N10	0.19775 (10)	0.3032 (3)	0.14935 (8)	0.0539 (6)	
C11	0.09265 (12)	0.1981 (3)	0.10599 (9)	0.0471 (6)	
C12	0.14047 (13)	0.2230 (3)	0.15271 (9)	0.0508 (6)	
C13	0.16483 (12)	0.3344 (3)	0.05474 (9)	0.0475 (6)	
C14	0.21142 (11)	0.3595 (3)	0.10230 (9)	0.0503 (6)	
C15	0.05947 (12)	0.2166 (3)	0.00717 (9)	0.0465 (6)	
O16	0.00735 (8)	0.3201 (2)	0.00150 (6)	0.0547 (5)	
O17	0.06869 (8)	0.1103 (2)	−0.02370 (6)	0.0632 (5)	
C18	−0.03879 (12)	0.3069 (3)	−0.04620 (9)	0.0463 (6)	
C19	−0.02424 (12)	0.3744 (3)	−0.09170 (9)	0.0544 (6)	
H19	0.0161	0.4252	−0.0917	0.065*	
C20	−0.07040 (12)	0.3656 (3)	−0.13730 (9)	0.0584 (7)	
H20	−0.0619	0.4093	−0.1690	0.070*	
C21	−0.12898 (12)	0.2915 (3)	−0.13526 (10)	0.0573 (7)	
C22	−0.14428 (12)	0.2281 (3)	−0.09000 (10)	0.0589 (7)	
H22	−0.1851	0.1800	−0.0900	0.071*	
C23	−0.09828 (12)	0.2366 (3)	−0.04435 (10)	0.0541 (6)	
H23	−0.1074	0.1952	−0.0126	0.065*	
F24	−0.17440 (7)	0.2840 (2)	−0.18049 (6)	0.0861 (5)	
C25	0.24602 (14)	0.3310 (4)	0.19848 (10)	0.0818 (9)	
H25A	0.2755	0.4207	0.1930	0.123*	
H25B	0.2702	0.2273	0.2079	0.123*	
H25C	0.2238	0.3629	0.2265	0.123*	
S26	0.15240 (4)	0.38413 (11)	0.35642 (3)	0.0719 (3)	
O27	0.15245 (13)	0.2321 (3)	0.32563 (8)	0.1034 (8)	
O28	0.11971 (11)	0.3678 (3)	0.40094 (7)	0.0928 (7)	
O29	0.21189 (9)	0.4779 (3)	0.36698 (9)	0.1031 (8)	
C30	0.09881 (15)	0.5223 (5)	0.31287 (13)	0.0770 (9)	
F31	0.11978 (10)	0.5488 (3)	0.26795 (7)	0.1135 (7)	
F32	0.09350 (10)	0.6752 (3)	0.33429 (9)	0.1138 (7)	
F33	0.03935 (9)	0.4604 (3)	0.30042 (8)	0.1163 (7)	

### Atomic displacement parameters (Å^2^)

**Table:**

	*U*^11^	*U*^22^	*U*^33^	*U*^12^	*U*^13^	*U*^23^
C1	0.0610 (18)	0.0645 (16)	0.0525 (16)	−0.0065 (14)	0.0053 (13)	−0.0087 (14)
C2	0.078 (2)	0.095 (2)	0.071 (2)	−0.0137 (17)	0.0241 (18)	−0.0010 (17)
C3	0.098 (3)	0.112 (3)	0.0518 (18)	−0.003 (2)	0.0229 (18)	0.0057 (17)
C4	0.077 (2)	0.092 (2)	0.0400 (16)	0.0092 (18)	0.0029 (14)	−0.0005 (15)
C5	0.0481 (16)	0.0763 (19)	0.0671 (19)	−0.0031 (14)	−0.0056 (14)	−0.0100 (16)
C6	0.0557 (18)	0.079 (2)	0.086 (2)	−0.0064 (16)	0.0130 (17)	−0.0049 (18)
C7	0.0716 (19)	0.0736 (19)	0.0665 (18)	−0.0049 (16)	0.0190 (16)	0.0019 (15)
C8	0.0601 (17)	0.0600 (16)	0.0462 (15)	−0.0010 (14)	0.0000 (12)	0.0017 (12)
C9	0.0509 (15)	0.0377 (12)	0.0391 (14)	0.0074 (11)	−0.0028 (11)	−0.0044 (10)
N10	0.0524 (14)	0.0609 (13)	0.0419 (13)	0.0078 (11)	−0.0074 (10)	−0.0062 (10)
C11	0.0519 (15)	0.0430 (13)	0.0439 (15)	0.0066 (12)	0.0026 (12)	−0.0042 (11)
C12	0.0584 (17)	0.0512 (15)	0.0398 (15)	0.0114 (13)	0.0017 (12)	−0.0043 (11)
C13	0.0466 (15)	0.0447 (14)	0.0469 (15)	0.0063 (12)	−0.0016 (12)	−0.0054 (11)
C14	0.0462 (15)	0.0529 (15)	0.0466 (15)	0.0067 (12)	−0.0045 (12)	−0.0038 (12)
C15	0.0499 (15)	0.0449 (14)	0.0409 (14)	0.0005 (12)	−0.0011 (12)	0.0005 (11)
O16	0.0563 (10)	0.0565 (10)	0.0448 (9)	0.0131 (9)	−0.0070 (8)	−0.0087 (8)
O17	0.0652 (12)	0.0623 (11)	0.0540 (11)	0.0152 (9)	−0.0090 (9)	−0.0200 (9)
C18	0.0475 (15)	0.0457 (13)	0.0406 (14)	0.0093 (12)	−0.0047 (11)	−0.0024 (11)
C19	0.0432 (14)	0.0631 (16)	0.0541 (16)	−0.0029 (12)	0.0016 (12)	−0.0018 (13)
C20	0.0553 (17)	0.0742 (17)	0.0432 (14)	0.0011 (14)	0.0027 (13)	0.0031 (13)
C21	0.0463 (16)	0.0646 (17)	0.0527 (16)	0.0050 (13)	−0.0116 (13)	−0.0039 (13)
C22	0.0426 (15)	0.0630 (17)	0.0661 (19)	−0.0024 (13)	−0.0027 (14)	0.0048 (14)
C23	0.0532 (16)	0.0524 (15)	0.0571 (16)	0.0047 (13)	0.0111 (13)	0.0099 (12)
F24	0.0617 (10)	0.1201 (13)	0.0636 (10)	−0.0034 (9)	−0.0210 (8)	0.0016 (9)
C25	0.0658 (19)	0.120 (3)	0.0491 (16)	−0.0003 (18)	−0.0162 (14)	−0.0050 (16)
S26	0.0683 (5)	0.0884 (5)	0.0517 (4)	0.0159 (4)	−0.0074 (4)	−0.0033 (4)
O27	0.145 (2)	0.0928 (16)	0.0634 (13)	0.0384 (15)	−0.0047 (13)	−0.0118 (12)
O28	0.1093 (17)	0.1221 (18)	0.0467 (11)	0.0021 (14)	0.0142 (11)	0.0038 (11)
O29	0.0523 (12)	0.138 (2)	0.1089 (17)	0.0033 (13)	−0.0110 (11)	0.0050 (15)
C30	0.069 (2)	0.095 (3)	0.068 (2)	0.0111 (18)	0.0138 (17)	0.0039 (19)
F31	0.1171 (15)	0.1541 (19)	0.0703 (12)	0.0225 (13)	0.0202 (11)	0.0343 (12)
F32	0.1120 (16)	0.0935 (15)	0.1339 (17)	0.0282 (12)	0.0182 (13)	0.0020 (13)
F33	0.0566 (11)	0.164 (2)	0.1149 (15)	0.0014 (12)	−0.0169 (10)	0.0110 (14)

### Geometric parameters (Å, °)

**Table:**

C1—C2	1.340 (4)	C13—C14	1.423 (3)
C1—C11	1.415 (3)	C15—O17	1.188 (3)
C1—H1	0.9300	C15—O16	1.340 (3)
C2—C3	1.398 (4)	O16—C18	1.412 (3)
C2—H2	0.9300	C18—C23	1.366 (3)
C3—C4	1.344 (4)	C18—C19	1.368 (3)
C3—H3	0.9300	C19—C20	1.370 (3)
C4—C12	1.399 (4)	C19—H19	0.9300
C4—H4	0.9300	C20—C21	1.362 (4)
C5—C6	1.332 (4)	C20—H20	0.9300
C5—C14	1.412 (4)	C21—F24	1.355 (3)
C5—H5	0.9300	C21—C22	1.356 (3)
C6—C7	1.403 (4)	C22—C23	1.369 (3)
C6—H6	0.9300	C22—H22	0.9300
C7—C8	1.344 (3)	C23—H23	0.9300
C7—H7	0.9300	C25—H25A	0.9600
C8—C13	1.413 (3)	C25—H25B	0.9600
C8—H8	0.9300	C25—H25C	0.9600
C9—C13	1.388 (3)	S26—O29	1.422 (2)
C9—C11	1.391 (3)	S26—O27	1.427 (2)
C9—C15	1.501 (3)	S26—O28	1.444 (2)
N10—C14	1.366 (3)	S26—C30	1.787 (3)
N10—C12	1.366 (3)	C30—F33	1.313 (3)
N10—C25	1.474 (3)	C30—F31	1.325 (3)
C11—C12	1.421 (3)	C30—F32	1.328 (4)
			
C2—C1—C11	121.0 (2)	C5—C14—C13	118.1 (2)
C2—C1—H1	119.5	O17—C15—O16	125.4 (2)
C11—C1—H1	119.5	O17—C15—C9	123.3 (2)
C1—C2—C3	119.5 (3)	O16—C15—C9	111.3 (2)
C1—C2—H2	120.3	C15—O16—C18	117.11 (17)
C3—C2—H2	120.3	C23—C18—C19	122.3 (2)
C4—C3—C2	122.0 (3)	C23—C18—O16	118.3 (2)
C4—C3—H3	119.0	C19—C18—O16	119.3 (2)
C2—C3—H3	119.0	C18—C19—C20	118.5 (2)
C3—C4—C12	120.2 (3)	C18—C19—H19	120.7
C3—C4—H4	119.9	C20—C19—H19	120.7
C12—C4—H4	119.9	C21—C20—C19	118.6 (2)
C6—C5—C14	120.8 (2)	C21—C20—H20	120.7
C6—C5—H5	119.6	C19—C20—H20	120.7
C14—C5—H5	119.6	F24—C21—C22	118.7 (2)
C5—C6—C7	121.8 (3)	F24—C21—C20	118.2 (2)
C5—C6—H6	119.1	C22—C21—C20	123.1 (2)
C7—C6—H6	119.1	C21—C22—C23	118.4 (2)
C8—C7—C6	119.3 (3)	C21—C22—H22	120.8
C8—C7—H7	120.3	C23—C22—H22	120.8
C6—C7—H7	120.3	C18—C23—C22	118.9 (2)
C7—C8—C13	121.5 (2)	C18—C23—H23	120.5
C7—C8—H8	119.3	C22—C23—H23	120.5
C13—C8—H8	119.3	N10—C25—H25A	109.5
C13—C9—C11	121.6 (2)	N10—C25—H25B	109.5
C13—C9—C15	118.3 (2)	H25A—C25—H25B	109.5
C11—C9—C15	120.1 (2)	N10—C25—H25C	109.5
C14—N10—C12	122.33 (19)	H25A—C25—H25C	109.5
C14—N10—C25	119.2 (2)	H25B—C25—H25C	109.5
C12—N10—C25	118.5 (2)	O29—S26—O27	116.26 (16)
C9—C11—C1	122.8 (2)	O29—S26—O28	114.84 (13)
C9—C11—C12	118.6 (2)	O27—S26—O28	114.55 (15)
C1—C11—C12	118.6 (2)	O29—S26—C30	103.21 (15)
N10—C12—C4	121.9 (2)	O27—S26—C30	102.78 (15)
N10—C12—C11	119.5 (2)	O28—S26—C30	102.50 (14)
C4—C12—C11	118.6 (3)	F33—C30—F31	107.3 (2)
C9—C13—C8	123.0 (2)	F33—C30—F32	106.4 (3)
C9—C13—C14	118.5 (2)	F31—C30—F32	106.7 (3)
C8—C13—C14	118.4 (2)	F33—C30—S26	112.4 (2)
N10—C14—C5	122.3 (2)	F31—C30—S26	111.7 (2)
N10—C14—C13	119.5 (2)	F32—C30—S26	112.0 (2)
			
C11—C1—C2—C3	−0.9 (4)	C6—C5—C14—C13	1.7 (4)
C1—C2—C3—C4	0.7 (5)	C9—C13—C14—N10	0.1 (3)
C2—C3—C4—C12	0.4 (5)	C8—C13—C14—N10	177.6 (2)
C14—C5—C6—C7	−0.1 (4)	C9—C13—C14—C5	−179.5 (2)
C5—C6—C7—C8	−1.3 (4)	C8—C13—C14—C5	−2.0 (3)
C6—C7—C8—C13	1.1 (4)	C13—C9—C15—O17	71.6 (3)
C13—C9—C11—C1	178.1 (2)	C11—C9—C15—O17	−105.2 (3)
C15—C9—C11—C1	−5.2 (3)	C13—C9—C15—O16	−107.5 (2)
C13—C9—C11—C12	−1.5 (3)	C11—C9—C15—O16	75.7 (3)
C15—C9—C11—C12	175.2 (2)	O17—C15—O16—C18	−3.1 (4)
C2—C1—C11—C9	−179.6 (2)	C9—C15—O16—C18	175.98 (19)
C2—C1—C11—C12	0.0 (4)	C15—O16—C18—C23	109.5 (2)
C14—N10—C12—C4	−179.9 (2)	C15—O16—C18—C19	−74.8 (3)
C25—N10—C12—C4	−0.5 (4)	C23—C18—C19—C20	−2.3 (4)
C14—N10—C12—C11	−0.7 (3)	O16—C18—C19—C20	−177.8 (2)
C25—N10—C12—C11	178.7 (2)	C18—C19—C20—C21	0.6 (4)
C3—C4—C12—N10	177.9 (3)	C19—C20—C21—F24	179.8 (2)
C3—C4—C12—C11	−1.3 (4)	C19—C20—C21—C22	1.0 (4)
C9—C11—C12—N10	1.5 (3)	F24—C21—C22—C23	−179.8 (2)
C1—C11—C12—N10	−178.2 (2)	C20—C21—C22—C23	−1.1 (4)
C9—C11—C12—C4	−179.3 (2)	C19—C18—C23—C22	2.3 (4)
C1—C11—C12—C4	1.1 (3)	O16—C18—C23—C22	177.9 (2)
C11—C9—C13—C8	−176.6 (2)	C21—C22—C23—C18	−0.6 (4)
C15—C9—C13—C8	6.6 (3)	O29—S26—C30—F33	−176.9 (2)
C11—C9—C13—C14	0.8 (3)	O27—S26—C30—F33	61.8 (3)
C15—C9—C13—C14	−176.0 (2)	O28—S26—C30—F33	−57.3 (3)
C7—C8—C13—C9	178.0 (2)	O29—S26—C30—F31	62.5 (3)
C7—C8—C13—C14	0.6 (4)	O27—S26—C30—F31	−58.8 (3)
C12—N10—C14—C5	179.4 (2)	O28—S26—C30—F31	−177.9 (2)
C25—N10—C14—C5	0.1 (4)	O29—S26—C30—F32	−57.1 (3)
C12—N10—C14—C13	−0.1 (3)	O27—S26—C30—F32	−178.4 (2)
C25—N10—C14—C13	−179.5 (2)	O28—S26—C30—F32	62.5 (3)
C6—C5—C14—N10	−177.8 (2)		

### Hydrogen-bond geometry (Å, °)

**Table:**

*D*—H···*A*	*D*—H	H···*A*	*D*···*A*	*D*—H···*A*
C1—H1···O17^i^	0.93	2.49	3.299 (3)	146
C4—H4···O27	0.93	2.46	3.185 (3)	134
C5—H5···O27^ii^	0.93	2.53	3.200 (4)	130
C22—H22···O29^iii^	0.93	2.54	3.399 (3)	153

Symmetry codes:  (i) −*x*, −*y*, −*z*; (ii) −*x*+1/2, *y*+1/2, −*z*+1/2; (iii) *x*−1/2, −*y*+1/2, *z*−1/2.

### Table 2 C-F···π and S-O···π interactions (Å,°).

**Table:**

X	*I*	*J*	*I*···*J*	X···*J*	X-*I*···*J*
C21	F24	*Cg*2^i^	3.870 (2)	3.616 (3)	69.12 (12)
C30	F33	*Cg*2^iv^	3.835 (2)	4.951 (4)	143.41 (19)
S26	O29	*Cg*1^ii^	3.646 (2)	5.055 (15)	170.66 (13)

Symmetry codes: (i) -*x*, -*y*, -*z*; (ii) -*x* + 1/2, *y* + 1/2, -*z* + 1/2; (iv) -*x*, *y*, -*z* + 1/2.Notes: *Cg*1 and *Cg*2 are the centroids of the C9/N10/C11-C14 and C1-C4/C11/C12 rings, respectively.

### Table 3 π-π interactions (Å,°).

**Table:**

*I*	*J*	*CgI*···*CgJ*	Dihedral angle	*CgI*_Perp	Cg*I*_Offset
1	4^v^	3.572 (2)	5.04 (11)	3.408 (1)	1.089 (2)
2	4^i^	3.856 (2)	4.29 (13)	3.596 (2)	1.392 (2)
3	4^v^	3.898 (2)	4.66 (12)	3.380 (2)	1.942 (2)
4	1^v^	3.572 (2)	5.04 (11)	3.472 (1)	0.839 (2)
4	2^i^	3.856 (2)	4.29 (13)	3.502 (1)	1.614 (2)
4	3^v^	3.898 (2)	4.66 (12)	3.483 (1)	1.750 (2)

Symmetry codes: (i) -*x*, -*y*, -*z*; (v) -*x*, -*y* + 1, -*z*.Notes: *Cg*1, *Cg*2, *Cg*3 and *Cg*4 are the centroids of the C9/N10/C11-C14, C1-C4/C11/C12, C5-C8/C13/C14 and C18-C23 rings, respectively. Cg*I*···Cg*J* is the distance between ring centroids. The dihedral angle is that between the planes of the rings *I* and *J*. *CgI*_Perp is the perpendicular distance of *CgI* from ring *J*. Cg*I*_Offset is the distance between *CgI* and perpendicular projection of *CgJ* on ring *I*.
